# Roles of USP1 in Ewing sarcoma

**DOI:** 10.18632/genesandcancer.235

**Published:** 2024-02-02

**Authors:** Panneerselvam Jayabal, Xiuye Ma, Yuzuru Shiio

**Affiliations:** ^1^Greehey Children’s Cancer Research Institute, The University of Texas Health Science Center, San Antonio, TX 78229, USA; ^2^Mays Cancer Center, The University of Texas Health Science Center, San Antonio, TX 78229, USA; ^3^Department of Biochemistry and Structural Biology, The University of Texas Health Science Center, San Antonio, TX 78229, USA

**Keywords:** chemotherapy, Ewing sarcoma, growth, USP1

## Abstract

Ewing sarcoma is a cancer of bone and soft tissue in children and young adults that is driven by the EWS-ETS fusion transcription factor, most commonly EWS-FLI1. We previously reported that Ewing sarcoma harbors two populations of cells, the CD133^high^ population displaying higher growth rate and the CD133^low^ population displaying chemotherapy resistance. We now find that the ubiquitin-specific protease 1 (USP1) is a transcriptional target of the EWS-FLI1 fusion oncoprotein, expressed at high and low levels in the CD133^high^ and the CD133^low^ populations, respectively, and determines chemo-sensitivity. We also find that USP1 inhibits cdc42, increases EWS-FLI1 transcriptional output, and simulates Ewing sarcoma growth. We show that chemo-sensitization by USP1 is independent of cdc42. A pharmacological inhibitor of USP1 was able to activate cdc42 and inhibit Ewing sarcoma growth. These results uncover critical roles for USP1 in Ewing sarcoma, which regulates growth and chemo-sensitivity via distinct mechanisms.

## INTRODUCTION

Ewing sarcoma is an aggressive bone and soft tissue cancer in children that is characterized by a chromosomal translocation between EWS and an Ets family transcription factor, most commonly FLI1 [1–3]. EWS-FLI1 translocation accounts for 85% of Ewing sarcoma cases. The EWS-FLI-1 gene product functions as an oncogenic transcription factor [1–3], recruiting the BAF chromatin remodeling complexes to its target genes [4].

We recently reported that Ewing sarcoma depends on the autocrine signaling mediated by a cytokine, NELL2 [5]. NELL2 binds to a receptor, Robo3, and stimulates the EWS-FLI1 transcriptional output through inactivation of cdc42, which disassembles and destabilizes the BAF complexes [5]. NELL2, CD133, and EWS-FLI1 positively regulate each other and upregulate the BAF complexes and cell proliferation in Ewing sarcoma [5]. We identified two populations of cells in Ewing sarcoma, NELL2^high^ CD133^high^ EWS-FLI1^high^ and NELL2^low^ CD133^low^ EWS-FLI1^low^, which display phenotypes consistent with high and low NELL2 signaling, respectively [5]. The CD133^high^ population displays higher growth rate while the CD133^low^ population grows more slowly and is more resistant to chemotherapy [5]. The molecular basis of differential chemotherapy sensitivity of the CD133^high^ and CD133^low^ populations remained unclear.

Ubiquitin-specific protease 1 (USP1) is a deubiquitinating enzyme that plays important roles in DNA damage response. USP1 is a key regulator of the Fanconi anemia pathway which repairs DNA interstrand crosslinks [6–8]. In response to DNA interstrand crosslinks, eight Fanconi anemia proteins assemble into a nuclear E3 ubiquitin ligase complex, which mono-ubiquitinates FANCD2 and FANCI. Mono-ubiquitinated FANCD2/I in turn serve as a platform to recruit factors involved in the repair of DNA interstrand crosslinks. USP1 deubiquitinates mono-ubiquitinated FANCD2/I, thereby reversing the critical step in the activation of the Fanconi anemia pathway [6–8]. USP1 also regulates translesion synthesis (TLS), in which specific DNA polymerases bypass DNA lesions which otherwise stall replication, by de-ubiquitinating mono-ubiquitinated PCNA (Proliferating Cell Nuclear Antigen) which promotes recruitment of TLS polymerases [9]. Other reported deubiquitination substrates/targets of USP1 include inhibitors of DNA binding (ID) proteins [10], ULK1 [11], Snail [12], KPNA2 [13], TAZ [14], KDM4A [15], Akt [16], TBLR1 [17], Estrogen Receptor alpha [18], RPS16 [19], SIX1 [20], and survivin [21, 22].

Upon further investigation of the mechanism of differential chemotherapy sensitivity of the CD133^high^ and CD133^low^ populations, we found that USP1 is a transcriptional activation target of EWS-FLI1 and is a key determinant of chemotherapy sensitivity in Ewing sarcoma. High and low expression of USP1 in the CD133^high^ and CD133^low^ populations, respectively, are responsible for chemosensitivity and chemoresistance of the CD133^high^ and CD133^low^ populations, respectively. We also found that USP1 inhibits cdc42, enhances EWS-FLI1 transcriptional output, and stimulates Ewing sarcoma growth. Interestingly, chemo-sensitization by USP1 is independent of cdc42. We demonstrate that a USP1 inhibitor, ML323, is able to activate cdc42 and inhibit growth in Ewing sarcoma.

## RESULTS AND DISCUSSION

### USP1 is a transcriptional target of EWS-FLI1

**Figure 1:**
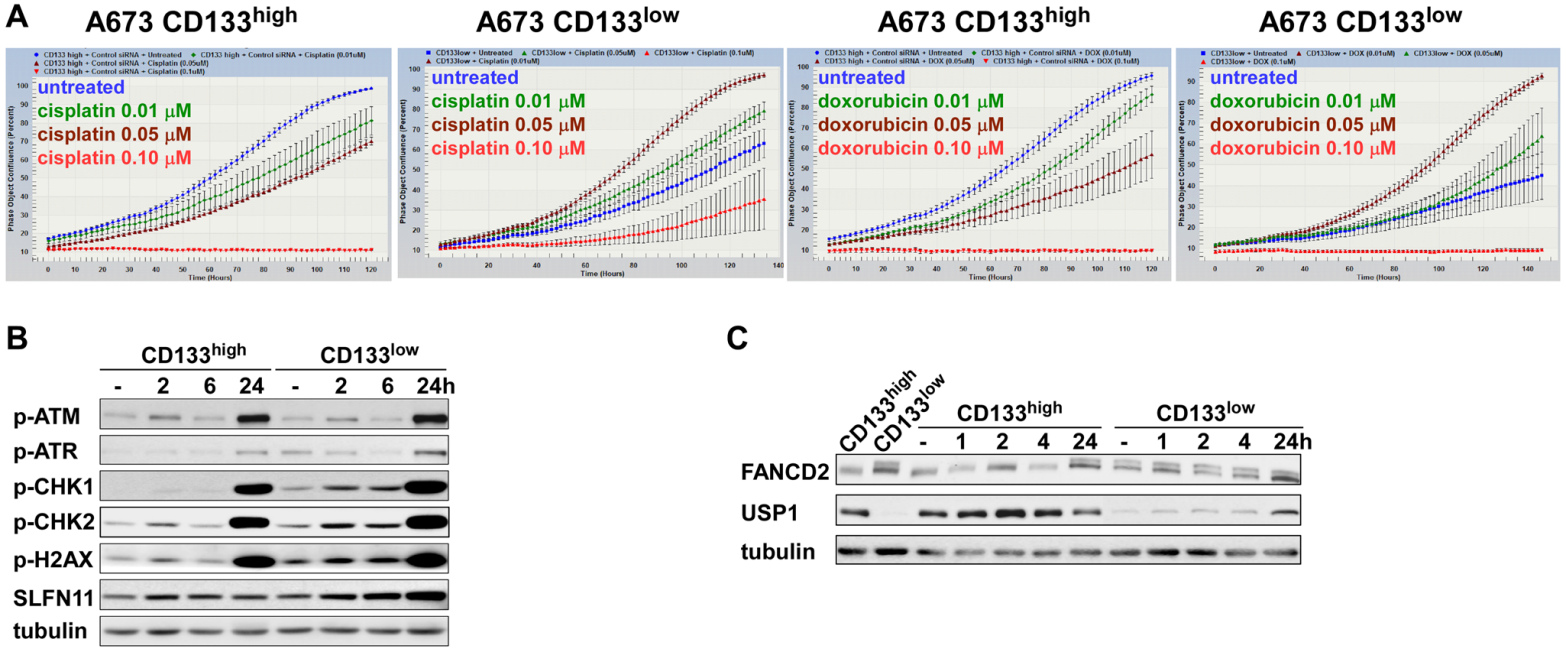
The CD133^low^ population of Ewing sarcoma is chemotherapy resistant. (**A**) A673 cells were sorted into the CD133^high^ and CD133^low^ populations. Each cell population was analyzed for the sensitivity to indicated concentration of cisplatin or doxorubicin using the IncuCyte live-cell imaging system. (**B**) The protein levels of the mediators of DNA damage response in the CD133^high^ and CD133^low^ populations. The CD133^high^ and CD133^low^ populations derived from A673 cells were left untreated or treated with 0.01 μM cisplatin for the indicated time and the levels of phosphorylated ATM, ATR, CHK1, CHK2, and H2AX as well as the levels of SLFN11 were examined by immunoblotting. Tubulin serves as a loading control. (**C**) The CD133^low^ population displays increased FANCD2 mono-ubiquitination and reduced USP1 levels compared with the CD133^high^ population. The CD133^high^ and CD133^low^ populations derived from A673 cells were left untreated or treated with 0.01 μM cisplatin for the indicated time and the levels of FANCD2 mono-ubiquitination and USP1 were examined by immunoblotting. Tubulin serves as a loading control.

We have reported that Ewing sarcoma harbors two populations of cells, NELL2^high^ CD133^high^ EWS-FLI1^high^ and NELL2^low^ CD133^low^ EWS-FLI1^low^ [5]. The CD133^high^ population grows more rapidly while the CD133^low^ population is more resistant to chemotherapy ([5] and Figure 1A). To gain an insight into the mechanism of differential chemotherapy sensitivity of the CD133^high^ and CD133^low^ populations, we assessed the levels of mediators of DNA damage response. While the levels of phosphorylated ATM, ATR, CHK1, CHK2, and H2AX as well as the levels of SLFN11, a key regulator of chemotherapy sensitivity in Ewing sarcoma and other cancers [23, 24], were similar in the two populations (Figure 1B), we found high levels of mono-ubiquitinated FANCD2 in the CD133^low^ population even in the absence of exogenous DNA damage (Figure 1C). Mono-ubiquitination of FANCD2 is normally removed by a de-ubiquitinating enzyme, USP1 [6]. We found that the CD133^low^ population displays low levels of USP1 compared with the CD133^high^ population (Figure 1C).

**Figure 2:**
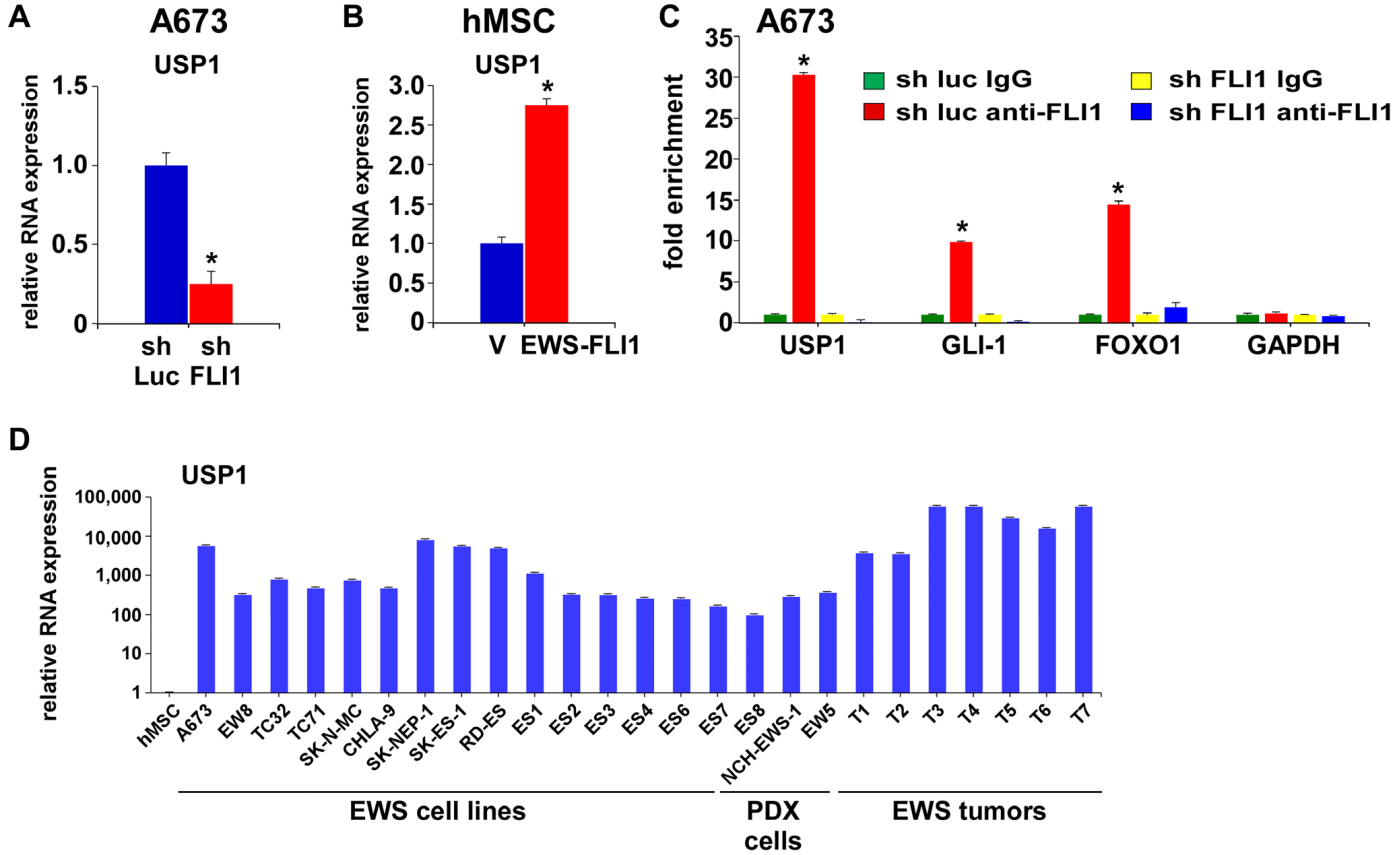
USP1 is a transcriptional target of EWS-FLI1. (**A**) EWS-FLI1 knockdown results in reduced USP1 transcript levels in A673 cells. ^*^p < 0.05. (**B**) EWS-FLI1 induces USP1 RNA expression in human mesenchymal stem cells. ^*^p < 0.05. (**C**) EWS-FLI1 binds to the USP1 gene promoter. Chromatin immunoprecipitation analysis for EWS-FLI1 binding to the promoter of USP1 and known EWS-FLI1 target genes (GLI-1 and FOXO1), as well as control (GAPDH), with and without EWS-FLI1 silencing in A673 cells. ^*^p < 0.05 compared with IgG chromatin immunoprecipitation of luciferase shRNA-expressing cells. (**D**) USP1 is highly expressed in Ewing sarcoma. The USP1 RNA expression in sixteen Ewing sarcoma cell lines, two Ewing sarcoma PDX tumor-derived cells, seven Ewing sarcoma tumor samples, and human mesenchymal stem cells was examined by qRT-PCR. All RNA levels were normalized to the RNA levels in human mesenchymal stem cells.

Because the CD133^low^ population displays diminished transcriptional activity of EWS-FLI1 compared with the CD133^high^ population [5], we tested a possibility that EWS-FLI1 regulates USP1 expression. Silencing of EWS-FLI1 in A673 Ewing sarcoma cells resulted in reduced USP1 transcript levels (Figure 2A) while exogenous expression of EWS-FLI1 in human mesenchymal stem cells, putative cells of origin of Ewing sarcoma [1–3], induced USP1 transcript levels (Figure 2B). Using chromatin immunoprecipitation, we were able to show that endogenous EWS-FLI1 in A673 Ewing sarcoma cells directly binds to the USP1 gene promoter (Figure 2C). A previous ChIP – sequencing study also identified USP1 as one of 1785 high-confidence EWS-FLI1-binding sites in A673 and SK-N-MC cells [25]. These results suggest that USP1 is a direct transcriptional target of EWS-FLI1. Consistent with this, USP1 was highly expressed in Ewing sarcoma tumors, patient-derived xenograft (PDX) tumor-derived cells, and cell lines compared with human mesenchymal stem cells (Figure 2D).

### USP1 determines chemotherapy sensitivity in Ewing sarcoma

**Figure 3:**
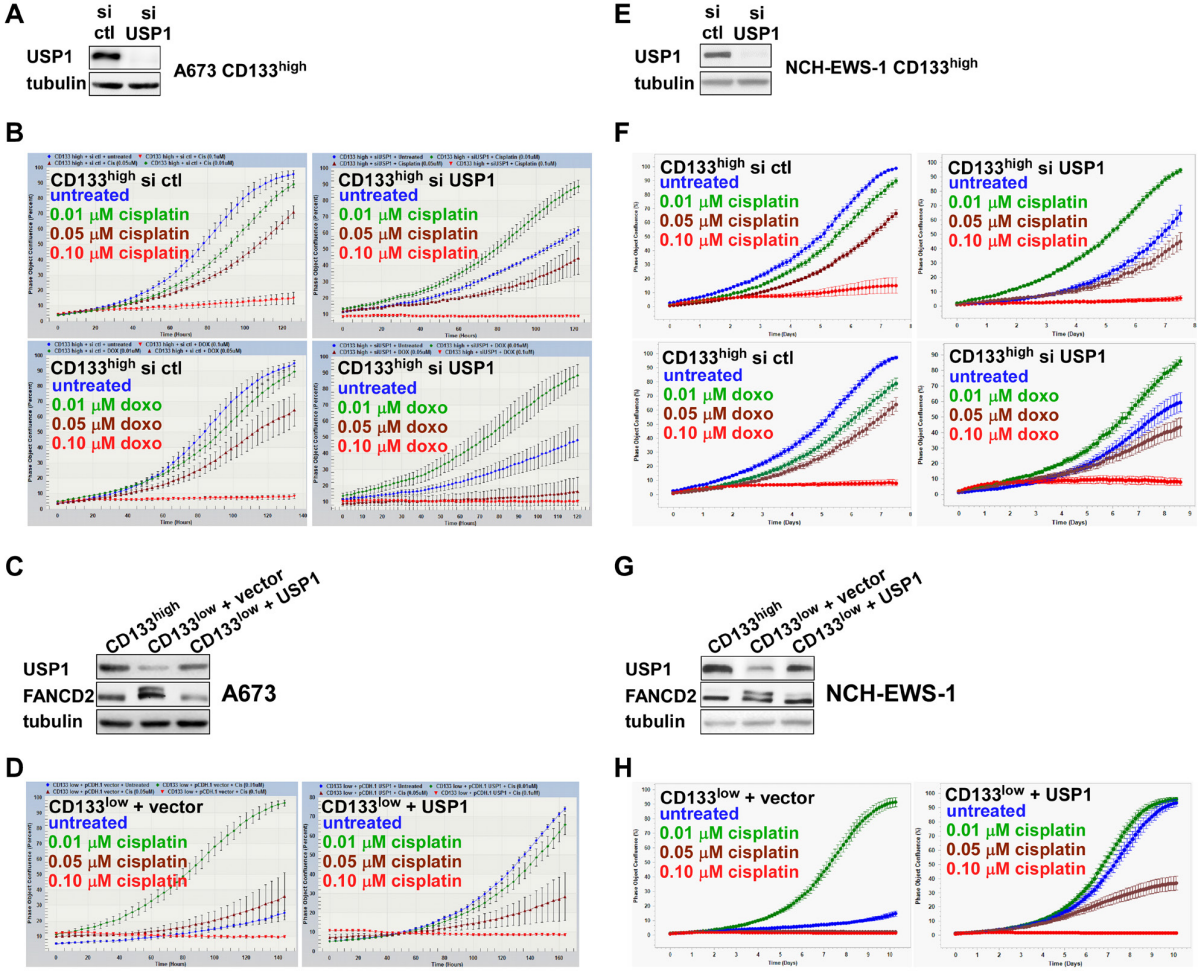
USP1 determines chemotherapy sensitivity in Ewing sarcoma. (**A**) USP1 knockdown in A673 CD133^high^ population. The CD133^high^ population derived from A673 cells was transfected with control siRNA or USP1 siRNA and the USP1 protein levels were assessed by immunoblotting. (**B**) USP1 knockdown makes A673 CD133^high^ population more resistant to chemotherapy. Cells in (A) were assessed for the sensitivity to indicated concentration of cisplatin or doxorubicin using IncuCyte. (**C**) USP1 exogenous expression in A673 CD133^low^ population. The CD133^low^ population derived from A673 cells was infected with lentiviruses expressing USP1 or empty vector and the levels of USP1 and FANCD2 were assessed by immunoblotting. (**D**) USP1 exogenous expression makes A673 CD133^low^ population more sensitive to chemotherapy. Cells in (C) were assessed for the sensitivity to indicated concentration of cisplatin using IncuCyte. (**E**) USP1 knockdown in NCH-EWS-1 CD133^high^ population. The CD133^high^ population derived from NCH-EWS-1 PDX tumor cells was transfected with control siRNA or USP1 siRNA and the USP1 protein levels were assessed by immunoblotting. (**F**) USP1 knockdown makes NCH-EWS-1 CD133^high^ population more resistant to chemotherapy. Cells in (E) were assessed for the sensitivity to indicated concentration of cisplatin or doxorubicin using IncuCyte. (**G**) USP1 exogenous expression in NCH-EWS-1 CD133^low^ population. The CD133^low^ population derived from NCH-EWS-1 PDX tumor cells was infected with lentiviruses expressing USP1 or empty vector and the levels of USP1 and FANCD2 were assessed by immunoblotting. (**H**) USP1 exogenous expression makes NCH-EWS-1 CD133^low^ population more sensitive to chemotherapy. Cells in (G) were assessed for the sensitivity to indicated concentration of cisplatin using IncuCyte.

USP1 is an important regulator of the DNA damage response, deubiquitinating mono-ubiquitinated FANCD2/I and PCNA [6, 8, 9]. We therefore examined the role of USP1 in differential chemotherapy sensitivity of the CD133^high^ and the CD133^low^ populations. While the CD133^high^ population of A673, EW8, and TC32 Ewing sarcoma cells displayed dose-dependent growth inhibition by chemotherapy, silencing of USP1 inhibited cell proliferation and made cells more resistant to chemotherapy (Figure 3A, 3B and Supplementary Figure 1A, 1B, 1E, 1F). Conversely, while the C133^low^ population of A673, EW8, and TC32 cells was more resistant to chemotherapy, USP1 lentivirus infection of the CD133^low^ population, which increased the USP1 levels comparable to those in the CD133^high^ population, stimulated cell proliferation, and made cells more sensitive to chemotherapy (Figure 3C, 3D and Supplementary Figure 1C, 1D, 1G, 1H). To complement an established cell line, we also employed cells dissociated from a Ewing sarcoma PDX tumor (NCH-EWS-1). Using these PDX tumor-derived cells, we found that USP1 silencing in the CD133^high^ population inhibits cell proliferation and makes cells more resistant to chemotherapy (Figure 3E, 3F) and that USP1 lentivirus infection of the CD133^low^ population stimulates cell proliferation and makes cells more sensitive to chemotherapy (Figure 3G, 3H).

Collectively, these results suggest that USP1 determines chemotherapy sensitivity in Ewing sarcoma.

### USP1 inhibits cdc42 and enhances EWS-FLI1 transcriptional output in Ewing sarcoma

We have previously demonstrated the key role for cdc42 in suppression of Ewing sarcoma growth [5]. Active cdc42 disassembles the BAF chromatin remodeling complexes and inhibits EWS-FLI1 transcriptional program, leading to growth arrest of Ewing sarcoma [5]. Because USP1 silencing in the CD133^high^ population inhibited proliferation and USP1 exogenous expression in the CD133^low^ population stimulated proliferation, we tested a possibility that USP1 affects active cdc42 levels.

**Figure 4:**
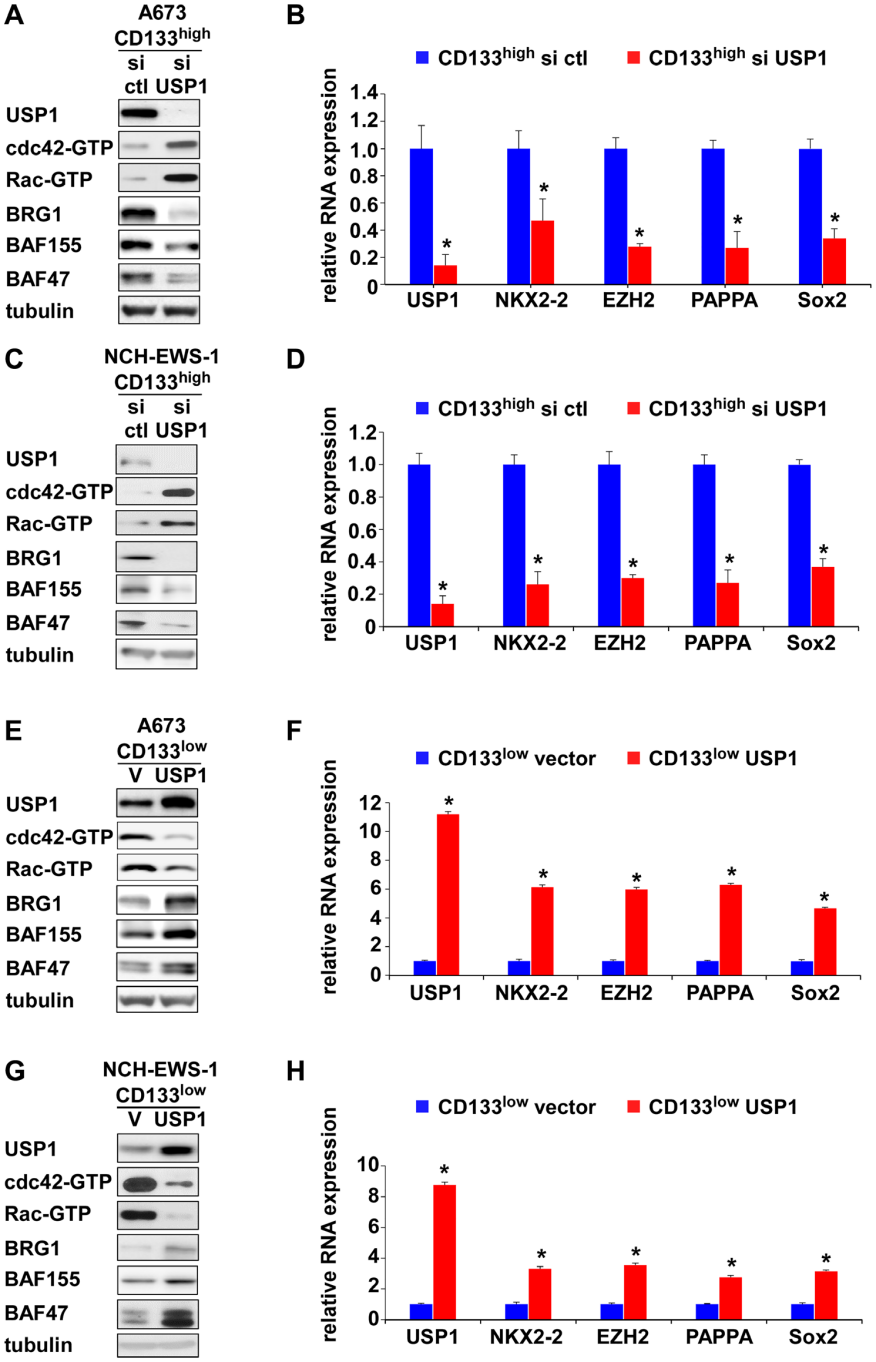
USP1 inhibits cdc42 and enhances EWS-FL1 transcriptional output. (**A**) USP1 knockdown in A673 CD133^high^ population results in activation of cdc42 and Rac and reduced BAF subunits. The levels of active cdc42 and active Rac were assessed by GST-PAK1 pulldown followed by immunoblotting. (**B**) USP1 knockdown in A673 CD133^high^ population results in reduced EWS-FLI1 target gene expression. NKX2-2, EZH2, PAPPA, and Sox2 are EWS-FLI1 target genes. ^*^p < 0.05. (**C**) USP1 knockdown in NCH-EWS-1 CD133^high^ population results in activation of cdc42 and Rac and reduced BAF subunits. (**D**) USP1 knockdown in NCH-EWS-1 CD133^high^ population results in reduced EWS-FLI1 target gene expression. ^*^p < 0.05. (**E**) USP1 exogenous expression in A673 CD133^low^ population results in inhibition of cdc42 and Rac and increased BAF subunits. (**F**) USP1 exogenous expression in A673 CD133^low^ population results in increased EWS-FLI1 target gene expression. ^*^p < 0.05. (**G**) USP1 exogenous expression in NCH-EWS-1 CD133^low^ population results in inhibition of cdc42 and Rac and increased BAF subunits. (**H**) USP1 exogenous expression in NCH-EWS-1 CD133^low^ population results in increased EWS-FLI1 target gene expression. ^*^p < 0.05.

USP1 silencing in the CD133^high^ population of A673, EW8, and TC32 cells and NCH-EWS-1 PDX tumor-derived cells increased active cdc42 as well as active Rac levels (Figure 4A, 4C and Supplementary Figure 2A, 2C), reduced BAF subunits, BRG1, BAF155, and BAF47 (Figure 4A, 4C and Supplementary Figure 2A, 2C) which are destabilized by active cdc42 [5], and suppressed the expression of EWS-FLI1 target genes (Figure 4B, 4D and Supplementary Figure 2B, 2D). Conversely, exogenous USP1 expression in the CD133^low^ population of A673, EW8, TC32, and NCH-EWS-1 cells decreased active cdc42 and active Rac levels (Figure 4E, 4G and Supplementary Figure 2E, 2G), increased BAF subunits, BRG1, BAF155, and BAF47 (Figure 4E, 4G and Supplementary Figure 2E, 2G), and induced the expression of EWS-FLI1 target genes (Figure 4F, 4H and Supplementary Figure 2F, 2H). Collectively, these results suggest that USP1 inhibits cdc42 and enhances EWS-FLI1 transcriptional output.

**Figure 5:**
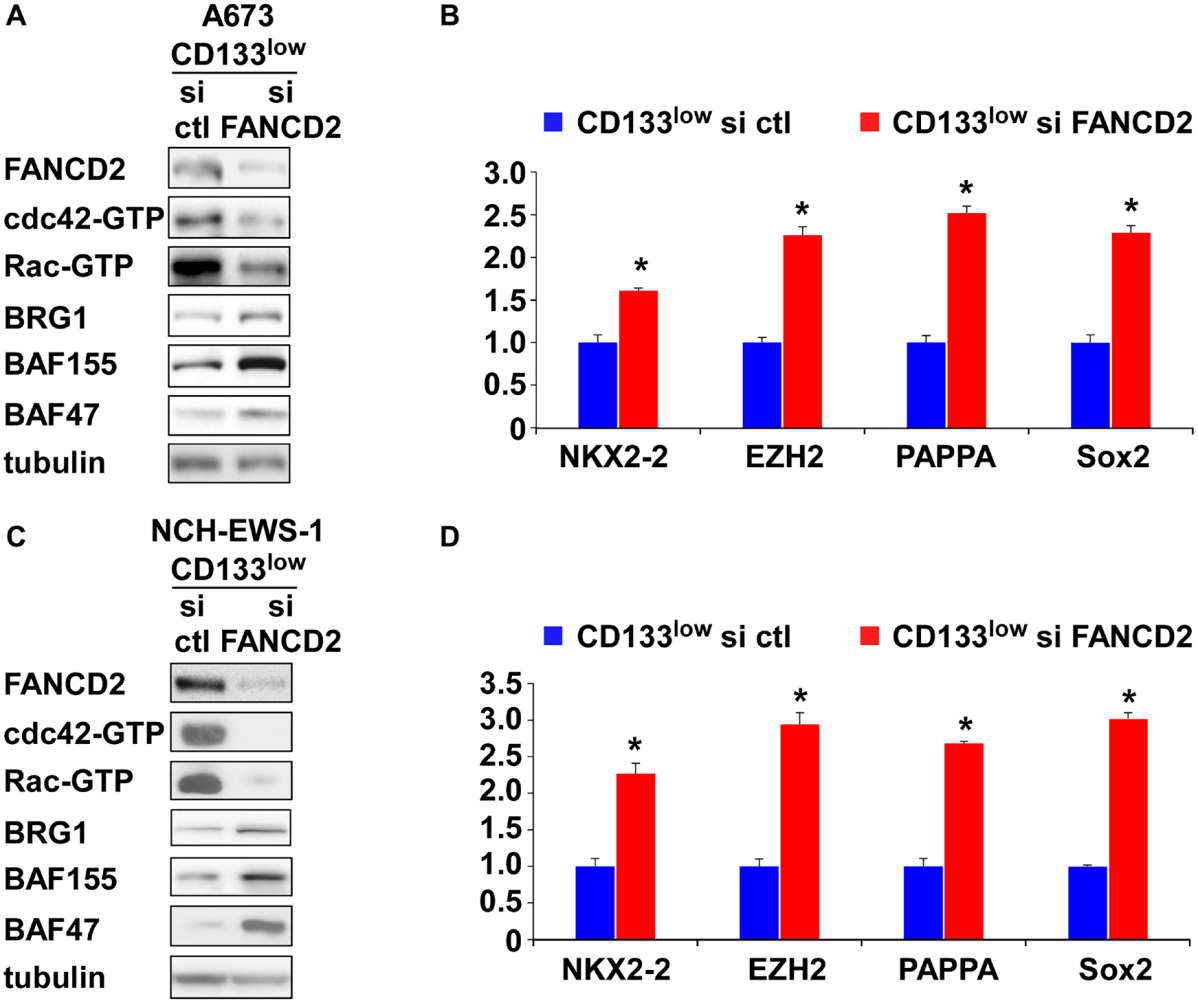
FANCD2 knockdown inhibits cdc42 and enhances EWS-FLI1 transcriptional output in the CD133^low^ population. (**A**) FANCD2 knockdown in A673 CD133^low^ population results in inhibition of cdc42 and Rac and increased BAF subunits. (**B**) FANCD2 knockdown in A673 CD133^low^ population results in increased EWS-FLI1 target gene expression. ^*^p < 0.05. (**C**) FANCD2 knockdown in NCH-EWS-1 CD133^low^ population results in inhibition of cdc42 and Rac and increased BAF subunits. (**D**) FANCD2 knockdown in NCH-EWS-1 CD133^low^ population results in increased EWS-FLI1 target gene expression. ^*^p < 0.05.

USP1 regulates the Fanconi anemia pathway by deubiquitinating mono-ubiquitinated FANCD2/I, which serve as a platform to recruit the factors that coordinate the repair of DNA interstrand crosslinks [7]. We therefore tested the effect of FANCD2 silencing on cdc42 activity in Ewing sarcoma. FANCD2 silencing in the CD133^low^ population of A673 cells and NCH-EWS-1 cells resulted in reduced active cdc42 as well as active Rac levels (Figure 5A, 5C), increased BAF subunits, BRG1, BAF155, and BAF47 (Figure 5A, 5C), and enhanced EWS-FLI1 target gene expression (Figure 5B, 5D), which phenocopies USP1 exogenous expression in the CD133^low^ populations (Figure 4E–4H and Supplementary Figure 2E–2H).

### USP1 inhibits cdc42 in a variety of non-ewing sarcoma cells as well

**Figure 6:**
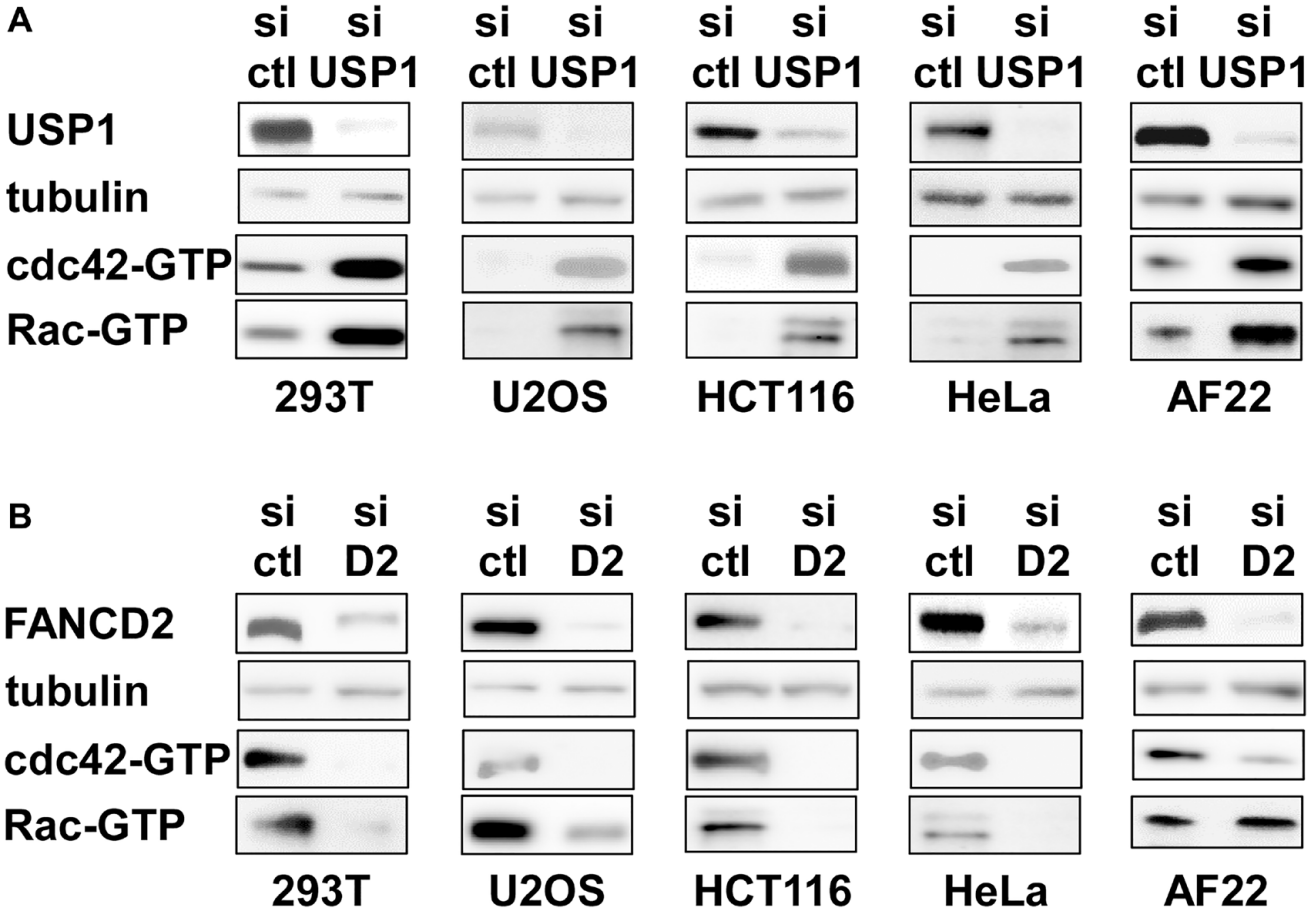
USP1 knockdown activates and FANCD2 knockdown inhibits cdc42 in non-Ewing sarcoma cells. (**A**) USP1 knockdown activates cdc42 in non-Ewing sarcoma cells. 293T, U2OS, HCT116, HeLa, and AF22 cells were transfected with control siRNA or USP1 siRNA and the levels of active cdc42 and active Rac were examined by GST-PAK1 pulldown assays. (**B**) FANCD2 knockdown inhibits cdc42 in non-Ewing sarcoma cells. 293T, U2OS, HCT116, HeLa, and AF22 cells were transfected with control siRNA or FANCD2 siRNA and the levels of active cdc42 and active Rac were examined by GST-PAK1 pulldown assays.

Regulation of cdc42 activity by USP1 and FANCD2 in Ewing sarcoma cells prompted us to test whether USP1 and FANCD2 also regulate cdc42 activity in non-Ewing sarcoma cells. USP1 silencing in 293T embryonic kidney cells, U2OS osteosarcoma cells, HCT116 colon cancer cells, HeLa cervical carcinoma cells, and AF22 neural stem cells resulted in increased active cdc42 as well as active Rac levels (Figure 6A). Conversely, FANCD2 silencing in these cells resulted in reduced active cdc42 and Rac levels, except for unaltered active Rac levels in AF22 cells (Figure 6B). These results suggest that USP1 – Fanconi anemia pathway regulates cdc42 activity in a variety of cell types. Stimulation of cdc42 activity by the Fanconi anemia pathway was previously suggested by reduced cdc42 activity in Fanconi anemia complementation group A patient bone marrow cells [26] and in mesenchymal stem cells derived from FANCA −/− or FANCC −/− mice [27].

### cdc42 does not determine chemotherapy sensitivity in Ewing sarcoma

**Figure 7:**
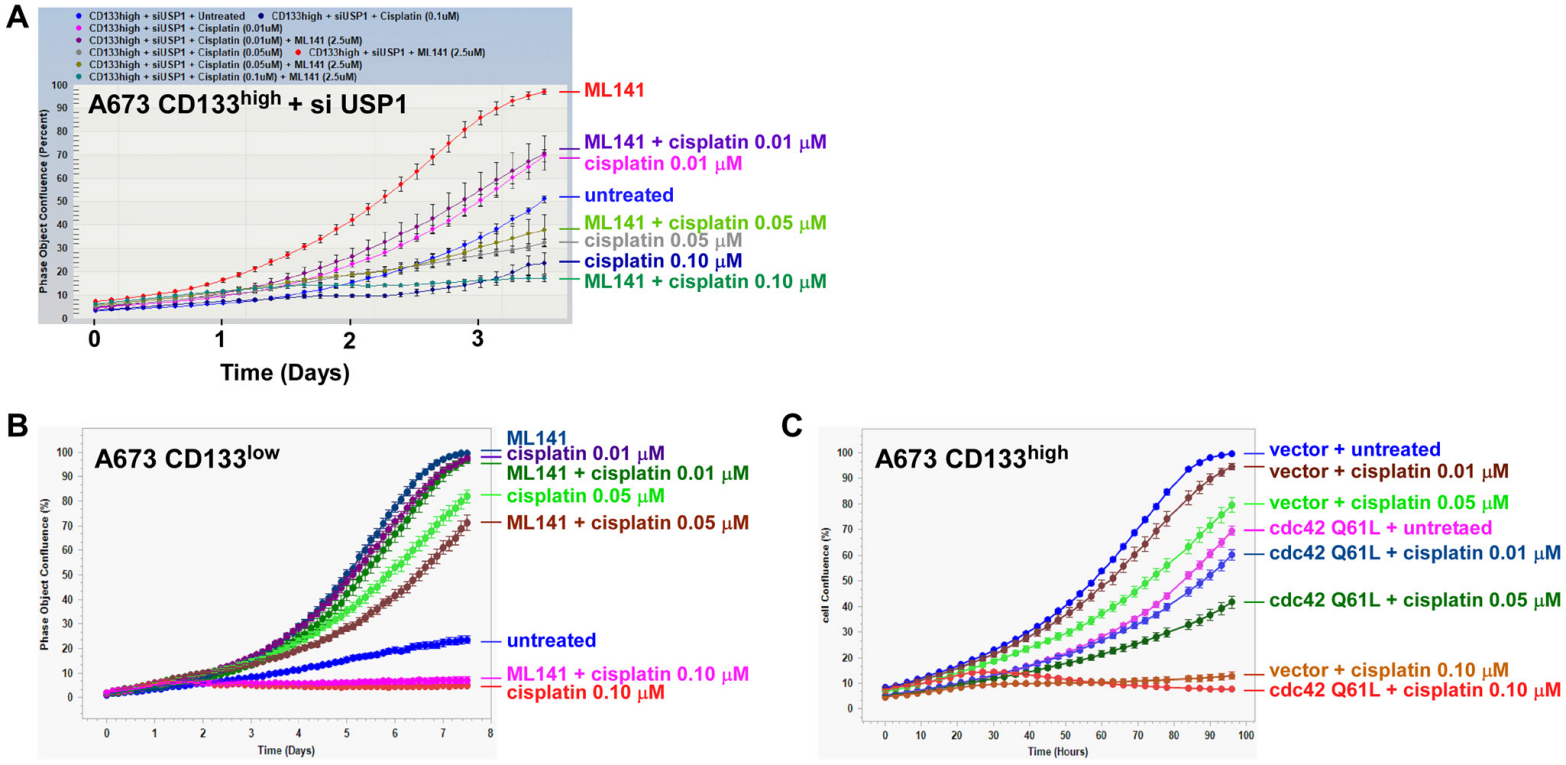
cdc42 does not determine chemotherapy sensitivity in Ewing sarcoma. (**A**) The effect of a cdc42 inhibitor, ML141, on chemotherapy sensitivity of the CD133^high^ population with USP1 knockdown. The CD133^high^ population derived from A673 cells was transfected with USP1 siRNA and was treated or not with 2.5 μM ML141. The sensitivity to indicated concentration of cisplatin was assessed using IncuCyte. (**B**) The effect of ML141 on chemotherapy sensitivity of the CD133^low^ population. The CD133^low^ population derived from A673 cells was treated or not with 2.5 μM ML141. The sensitivity to indicated concentration of cisplatin was assessed using IncuCyte. (**C**) The effect of cdc42 Q61L active mutant on chemotherapy sensitivity of the CD133^high^ population. The CD133^high^ population derived from A673 cells was infected with lentiviruses expressing cdc42 Q61L or empty vector. The sensitivity to indicated concentration of cisplatin was assessed using IncuCyte.

Inhibition of cdc42 by USP1 raised a possibility that USP1 may determine chemotherapy sensitivity of Ewing sarcoma through cdc42. USP1 silencing in the CD133^high^ population inhibited A673 cell proliferation (Figure 3B), which was rescued by a cdc42 inhibitor, ML141 (Figure 7A; untreated vs. ML141), suggesting that USP1 normally stimulates Ewing sarcoma growth through cdc42 inhibition. However, ML141 did not chemo-sensitize USP1-silenced CD133^high^ cells, and these cells displayed similar dose-dependent growth inhibition by cisplatin regardless of the presence of ML141 (Figure 7A). ML141 rescued the slow growth of the CD133^low^ population (Figure 7B and [5]), but ML141 did not chemo-sensitize the CD133^low^ population (Figure 7B; Compare with and without ML141 at each dose of cisplatin treatment). Lentiviral expression of constitutively active cdc42 Q61L mutant inhibited proliferation of CD133^high^ cells, but cdc42 Q61L-expressing CD133^high^ cells still displayed dose-dependent growth inhibition by cisplatin (Figure 7C). Collectively, these results suggest that while USP1 stimulates Ewing sarcoma growth through cdc42 inactivation, cdc42 does not mediate chemo-sensitization by USP1 in Ewing sarcoma.

### A USP1 inhibitor activates cdc42 and inhibits Ewing sarcoma growth

**Figure 8:**
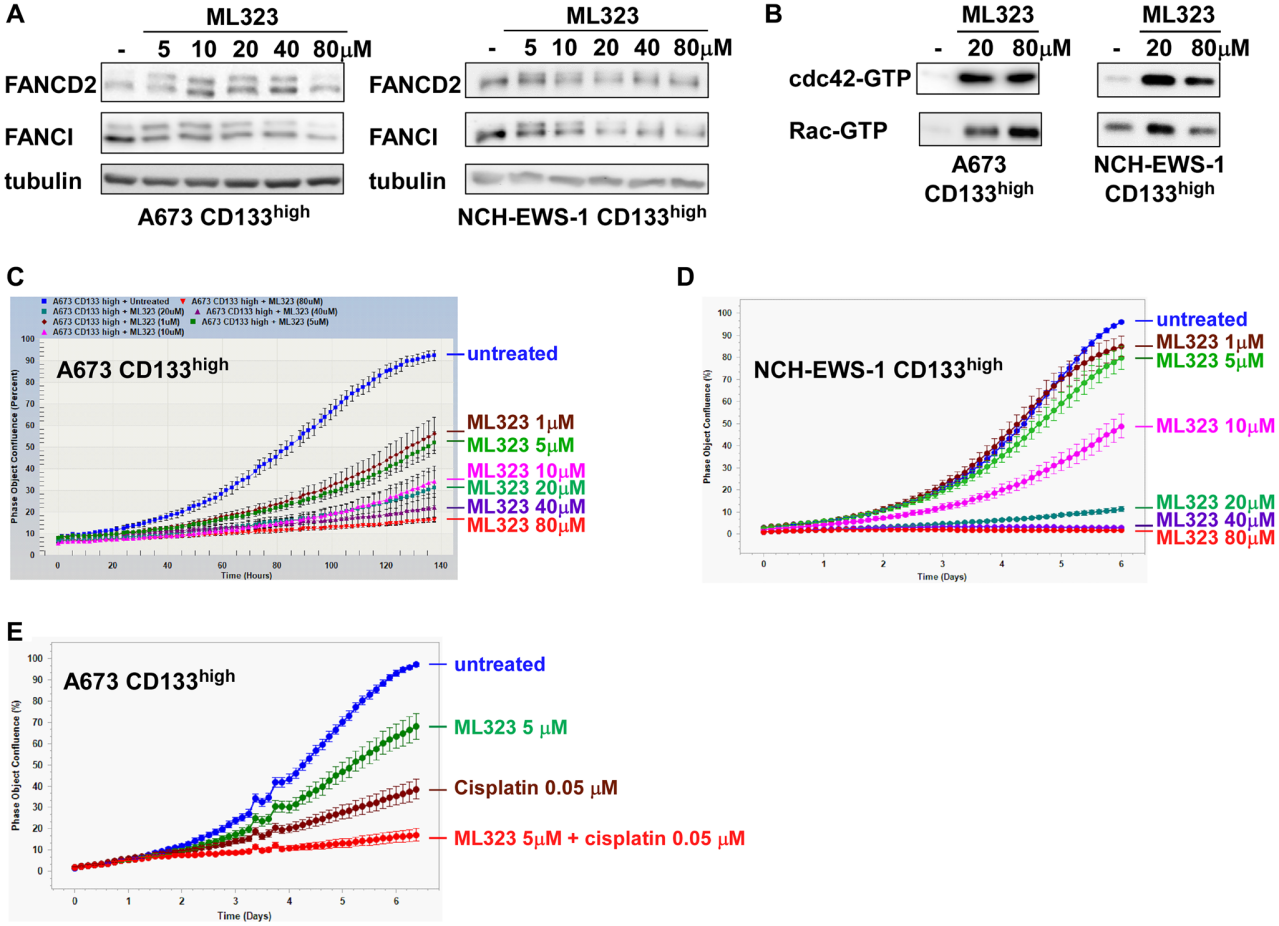
A USP1 inhibitor, ML323, activates cdc42 and inhibits Ewing sarcoma growth. (**A**) ML323 increases mono-ubiquitination of FANCD2 and FANCI. The CD133^high^ populations derived from A673 cells and NCH-EWS-1 PDX tumor-derived cells were treated with the indicated concentration of ML323 for 24 hours and FANCD2, FANCI, and USP1 protein expression was analyzed by immunoblotting. Tubulin serves as a loading control. (**B**) ML323 activates cdc42. A673 and NCH-EWS-1 CD133^high^ populations were treated with 0, 20, or 80 μM ML323 for 24 hours and the levels of active cdc42 and active Rac were examined by GST-PAK1 pulldown assays. (**C**) ML323 inhibits A673 CD133^high^ cell growth in a dose-dependent manner. A673 CD133^high^ cells were treated with the indicated concentration of ML323, and cell proliferation was assessed by IncuCyte. (**D**) ML323 inhibits NCH-EWS-1 CD133^high^ cell growth in a dose-dependent manner. NCH-EWS-1 CD133^high^ cells were treated with the indicated concentration of ML323, and cell proliferation was assessed by IncuCyte. (**E**) Synergistic growth inhibition of Ewing sarcoma cells by ML323 and cisplatin. A673 CD133^high^ cells were treated with 5 μM ML323 and/or 0.05 μM cisplatin, and cell proliferation was assessed by IncuCyte.

USP1-mediated regulation of cdc42 and growth in Ewing sarcoma led us to examine the effect of a pharmacological inhibitor of USP1, ML323 [28], on this cancer. ML323 treatment of the CD133^high^ population of A673 cells and NCH-EWS-1 PDX tumor-derived cells resulted in increased mono-ubiquitination of FANCD2 and FANCI as expected for the inhibition of USP1 deubiquitinating enzyme activity (Figure 8A). ML323 treatment also activated cdc42 as well as Rac in these Ewing sarcoma cells (Figure 8B), consistent with the inhibition of cdc42 and Rac by USP1 (Figure 4). ML323 also inhibited proliferation of A673 and NCH-EWS-1 CD133^high^ cells in a dose-dependent manner (Figure 8C, 8D). Furthermore, we observed synergy between ML323 and cisplatin in inhibiting Ewing sarcoma cell proliferation (Figure 8E). These results suggest that USP1 can be a potential therapeutic target in Ewing sarcoma. It will be important to further evaluate the anti-Ewing sarcoma effect of ML323 singly or in combination with chemotherapy, using both cell culture and mouse xenograft models.

This study uncovered important roles for USP1 in Ewing sarcoma. USP1 inhibits cdc42 and thereby stimulates growth in Ewing sarcoma. USP1 also determines chemotherapy sensitivity in Ewing sarcoma and this activity of USP1 is independent of cdc42. We previously identified cdc42 as a key regulator of growth in Ewing sarcoma [5]. Active cdc42 disassembles the BAF chromatin remodeling complexes and suppresses EWS-FLI1 transcriptional output, leading to growth arrest in Ewing sarcoma [5]. The data presented in this study suggest that USP1 inhibits cdc42 and maintains the BAF complex stability, EWS-FLI1 transcriptional output, and cell proliferation in Ewing sarcoma. The well-characterized function of USP1 is to deubiquitinate mono-ubiquitinated FANCD2/I, reversing the critical activation step of the Fanconi anemia pathway [6–8]. FANCD2 knockdown phenocopied USP1 exogenous expression in the CD133^low^ population of Ewing sarcoma (Figure 4E–4H and Figure 5). Furthermore, USP1 knockdown activated and FANCD2 knockdown inhibited cdc42 in multiple non-Ewing sarcoma cells (Figure 6), suggesting that the USP1 – Fanconi anemia pathway regulates cdc42 activity in a variety of cell types. Previous reports on reduced cdc42 activity in Fanconi anemia complementation group A patient bone marrow cells [26] and in mesenchymal stem cells derived from FANCA −/− or FANCC −/− mice [27] also suggest that the Fanconi anemia pathway stimulates cdc42 activity. While the molecular mechanism of cdc42 regulation by the USP1 – Fanconi anemia pathway is unclear, the emerging link to cdc42 may provide clues to elusive molecular events downstream of the Fanconi anemia pathway.

While this manuscript was in preparation, Mallard et al. reported the role of USP1 in Ewing sarcoma [22]. They found that USP1 is a transcriptional target of EWS-FLI1 and is highly expressed in Ewing sarcoma [22], which agrees well with our findings (Figure 2). They also reported that USP1 stabilizes survivin (BIRC5) and suppresses apoptosis in Ewing sarcoma [22]. Survivin was also reported by others as a deubiquitination substrate of USP1 [21]. We therefore assessed survivin protein levels and apoptosis induction upon USP1 manipulation in Ewing sarcoma cells and non-Ewing sarcoma cells. In our hands, USP1 knockdown did not consistently reduce survivin protein levels and induce apoptosis as judged by cleavage of caspase-3 and PARP in Ewing sarcoma cells and non-Ewing sarcoma cells (Supplementary Figure 3A, 3B, 3D). While the treatment of A673 CD133^high^ cells and NCH-EWS-1 CD133^high^ cells with high doses of ML323 did reduce survivin protein levels and induce some apoptotic changes, at lower doses, ML323 did not affect survivin protein levels and did not detectably induce apoptosis (Supplementary Figure 3C) although increased FANCD2/I mono-ubiquitination was evident at these lower doses (Figure 8A).

**Figure 9:**
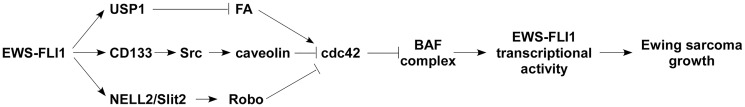
EWS-FLI1 stimulates multiple cdc42-inhibiting pathways in Ewing sarcoma.

We have reported multiple parallel signaling pathways that inhibit cdc42 and maintain Ewing sarcoma growth. These include NELL2 – Robo3 signaling [5], Slit2 – Robo1/2 signaling [29], and CD133 – Src – caveolin signaling [5]. The present study adds the USP1 – Fanconi anemia pathway to the list of cdc42-inhibiting pathways in Ewing sarcoma (Figure 9). Notably, USP1, NELL2, Slit2, and CD133 are all transcriptional targets of EWS-FLI1 (Figure 9). We propose that, by stimulating multiple cdc42-inhibiting pathways, EWS-FLI1 inactivates cdc42 and drives Ewing sarcoma.

## MATERIALS AND METHODS

### Cell culture

A673, SK-N-MC, 293T, U2OS, HeLa, and HCT116 cells were cultured in Dulbecco’s modified Eagle’s medium (DMEM) supplemented with 10% fetal bovine serum. EW8, TC32, TC71, CHLA-9, ES1, ES2, ES3, ES4, ES6, ES7, ES8, and RD-ES cells were cultured in RPMI-1640 medium supplemented with 10% fetal bovine serum. SK-NEP-1 and SK-ES-1 cells were cultured in McCoy’s 5a medium supplemented with 15% fetal bovine serum. NCH-EWS-1 cells dissociated from a Ewing sarcoma patient-derived xenograft tumor were cultured in DMEM/F-12 medium supplemented with 10% FBS [5]. AF22 cells were obtained from Dr. Anna Falk and cultured in DMEM/F-12 medium supplemented with 20% KSR, 10 μl/ml N-2 supplement (Thermo Fisher Scientific), 1 μl/ml B-27 supplement (Thermo Fisher Scientific), 10 ng/ml of bFGF (PeproTech), and 10 ng/ml of FGF (PeproTech), and the culture medium was replaced every second day. AF22 cells were plated on tissue culture plates coated with 0.1 mg/ml poly-L-ornithine (Sigma-Aldrich) and 10 μg/ml laminin (Sigma-Aldrich). A673, SK-N-MC, SK-NEP-1, SK-ES-1, RD-ES, RD, 293T, U2OS, HeLa, and HCT116 cells were from ATCC. TC71 cells were from the Coriell Institute for Medical Research. EW8, TC32, and CHLA-9 cells were from Dr. Patrick Grohar. The cell lines were STR-authenticated and were routinely tested for the absence of mycoplasma. Cord blood-derived human mesenchymal stem cells were purchased from Vitro Biopharma (Golden, CO) and were cultured in low-serum MSC-GRO following the manufacturer’s procedure. Calcium phosphate co-precipitation was used for transfection of 293T cells. Lentiviruses were prepared by transfection in 293T cells following System Biosciences’ protocol and the cells infected with lentiviruses were selected with 2 μg/ml puromycin for 48 hours as described [5, 30]. The target sequences for shRNAs are as follows: FLI1 C-terminus shRNA, AACGATCAGTAAGAATACAGAGC, USP1 shRNA #1, GACTGAATAATCTCGGCAATA, USP1 shRNA #2, CCAGAGACAAACTAGATCAAA, USP1 shRNA #3, CCAGTGACCAAACAGGCATTA, USP1 shRNA #4, GCTCGTATTTGTATTCTCCAT, USP1 shRNA #5, GCTAGTGGTTTGGAGTTTGAT, luciferase shRNA, GCACTCTGATTGACAAATACGATTT, and scrambled control shRNA, CCTAAGGTTAAGTCGCCCTCG. The following siRNAs were used: human USP1 siRNA SMARTpool (M-006061-02-0005; Dharmacon); human FANCD2 siRNA SMARTpool (M-016376-02-0010; Dharmacon), and Non-Targeting siRNA Pool #2 (D-001206-14-05; Dharmacon). siRNA transfection was performed using Lipofectamine™ RNAiMAX Transfection Reagent (Thermo Fisher). ML323 (18010) and cisplatin (13119) were from Cayman Chemical. Doxorubicin hydrochloride (D1515) was from MilliporeSigma. ML141(S7686) was from Selleck Chemicals.

### Flow cytometry

Cells were trypsinized, washed with FACS wash buffer (PBS, 0.5% BSA, 2 mM EDTA), and incubated with PE-conjugated human CD133/1 antibody (clone AC133, Miltenyi Biotec; 1:100 in FACS wash buffer) for 20 minutes at 4°C. Cells were washed three times with FACS wash buffer and the CD133^high^ and CD133^low^ cell populations were sorted by using BD FACSAria (Becton Dickinson). The FACSDiva 6.1.3 software (Becton Dickinson) was used for sample analysis.

### RNA samples and quantitative real-time PCR

Total cellular RNA was isolated using TRIzol reagent (Invitrogen). Reverse transcription was performed using a High Capacity cDNA Reverse Transcription Kit (Thermo Fisher) as per manufacturer’s instructions. Quantitative PCR was performed using PowerUp SYBR Green Master Mix (Thermo Fisher) on Applied Biosystems ViiA 7 Real-Time PCR System. Each sample was analyzed in triplicate. The following primers were used: USP1 forward, 5′-GCCACTCAGCCAAGGCGACTG-3′, USP1 reverse, 5′-CAGAATGCCTCATACTGTCCATCTCTATGC-3′; NKX2-2 forward, 5′-CAGCGACAACCCGTACAC-3′, NKX2-2 reverse, 5′-GACTTGGAGCTTGAGTCCTGA-3′; EZH2 forward, 5′-TGGGAAAGTACACGGGGATA-3′, EZH2 reverse, 5′-TATTGACCAAGGGCATTCAC-3′; PAPPA forward, 5′-CAGAATGCACTGTTACCTGGA-3′, PAPPA reverse, 5′-GCTGATCCCAATTCTCTTTCA-3′; Sox2 forward, 5′-GAGCTTTGCAGGAAGTTTGC-3′, Sox2 reverse, 5′-GCAAGAAGCCTCTCCTTGAA-3′; and GAPDH forward, 5′-GGTGTGAACCATGAGAAGTATGA-3′, GAPDH reverse, GAGTCCTTCCACGATACCAAAG.

### Immunoblotting

Fifteen μg of whole-cell lysate was separated by SDS-PAGE and analyzed by immunoblotting as described [5, 30]. The following primary antibodies were used: rabbit monoclonal anti-USP1 (8033, Cell Signaling Technologies); rabbit polyclonal anti-FANCD2 (NB100-182, Novus Biologicals); mouse monoclonal anti-FANCI (sc-271316, Santa Cruz Biotechnology); rabbit polyclonal anti-FLI1 (ab15289, Abcam); mouse monoclonal anti-FLAG M2 (F1804, MilliporeSigma); mouse monoclonal anti-cdc42 (ACD03, Cytoskeleton); rabbit polyclonal anti-Rac1/2/3 (2465, Cell Signaling Technologies); goat polyclonal anti-BRG1 (A303-877A, Bethyl Laboratories); rabbit monoclonal anti-BAF155 (11956, Cell Signaling Technologies); rabbit monoclonal anti-BAF47 (8745, Cell Signaling Technologies); rabbit monoclonal anti-phospho-ATM (Ser1981) (5883, Cell Signaling Technologies); rabbit monoclonal anti-phospho-ATR (Thr1989) (30632, Cell Signaling Technologies); rabbit monoclonal anti-phospho-Chk1 (Ser345) (2348, Cell Signaling Technologies); rabbit polyclonal anti-phospho-Chk2 (Thr68) (2661, Cell Signaling Technologies); mouse monoclonal anti-phospho-Histone H2A.X (Ser139) (05-636, MilliporeSigma); mouse monoclonal anti-Slfn11 (sc-374339, Santa Cruz Biotechnology); rabbit monoclonal anti-Survivin (2808, Cell Signaling Technologies); rabbit monoclonal anti-Caspase-3 (9665, Cell Signaling Technologies); rabbit polyclonal anti-PARP (9542, Cell Signaling Technologies); and mouse monoclonal anti-tubulin (DM1A, Thermo Fisher Scientific); The following HRP-conjugated secondary antibodies were used: goat anti-rabbit (7074) and goat anti-mouse (7076) (Cell Signaling Technologies) and donkey anti-goat (A50-201P, Bethyl Laboratories).

### Chromatin immunoprecipitation

Chromatin immunoprecipitation (ChIP) was performed as described [5] using rabbit polyclonal anti-FLI1 antibody (ab15289, Abcam) or control rabbit IgG (ab37415, Abcam). Cells were treated with 1% formaldehyde for 10 min at room temperature and cross-linking was quenched by 0.1375 M glycine. Harvested cells were lysed in cell lysis buffer (5 mM PIPES (pH 8.0), 85 mM KCl, 0.5% NP-40) and cell nuclei were pelleted. Cell nuclei were lysed in nuclei lysis buffer (50 mM Tris-Cl (pH 8.0), 10 mM EDTA, 1% SDS, protease inhibitors) and sonicated to generate DNA fragments between 300 and 1000 bp in size. DNA was eluted from immunoprecipitate using elution buffer (10 mM Tris-Cl (pH 8.0), 300 mM NaCl, 5 mM EDTA, 1% SDS) supplemented with proteinase K for 2 h at 55°C, followed by overnight incubation at 65°C. DNA in supernatant was purified by phenol-chloroform extraction and was used for qPCR. The PCR primer sequences were as follows:

USP1 forward, 5′-TCCCAAAGTGCTGGGATTAC-3′, USP1 reverse, 5′-ATGGATTAAGGGTCGCATTACA-3′; FOXO1 forward, 5′-GGAAGAGGTTCCCACGGAGGGCAT-3′, FOXO1 reverse, 5′-CCGGCGACACTTTGTTTACT-3′; GLI-1 forward, 5′-AGAGCCTGGGGGTGAGACAT-3′, GLI-1 reverse, 5′-GCCTCTTCAACTTAACCGCATGA-3′; and GAPDH forward, 5′-TCCTCCTGTTTCATCCAAGC-3′; GAPDH reverse, 5′-TAGTAGCCGGGCCCTACTTT-3′.

### GST pull-down assays

The active form of cdc42 and Rac was analyzed by pull-down of whole cell lysate with GST-PAK1 (which selectively binds active cdc42 and active Rac) followed by immunoblotting for cdc42 and Rac as described [31].

### Cell proliferation assays

Cell proliferation was assessed by the IncuCyte live-cell imaging system (Essen BioScience). The IncuCyte system monitors cell proliferation by analyzing the occupied area (% confluence) of cell images over time. At least four fields from four wells were assayed for each experimental condition. The cell seeding density was 2000 cells per well in a 96-well plate. For each assay, biological replicates were performed to confirm the reproducibility of results.

### Statistical analysis

Statistical analyses were performed with Prism (GraphPad Software, version 10.0.2) with a two-tailed Student’s *t* test. Data are expressed as mean ± SEM. The results were considered significant when *p* < 0.05.

## SUPPLEMENTARY MATERIALS


