# Supplementary material for: Roles of USP1 in Ewing sarcoma
Source: Genes Cancer. 2024 Feb 2;15:15–27. doi: 10.18632/genesandcancer.235 (PMC10843185; doi:10.18632/genesandcancer.235)
Supplement: Supplementary file 1 [file ganc-15-235-s001.pdf]

Roles of USP1 in Ewing sarcoma

SUPPLEMENTARY MATERIALS

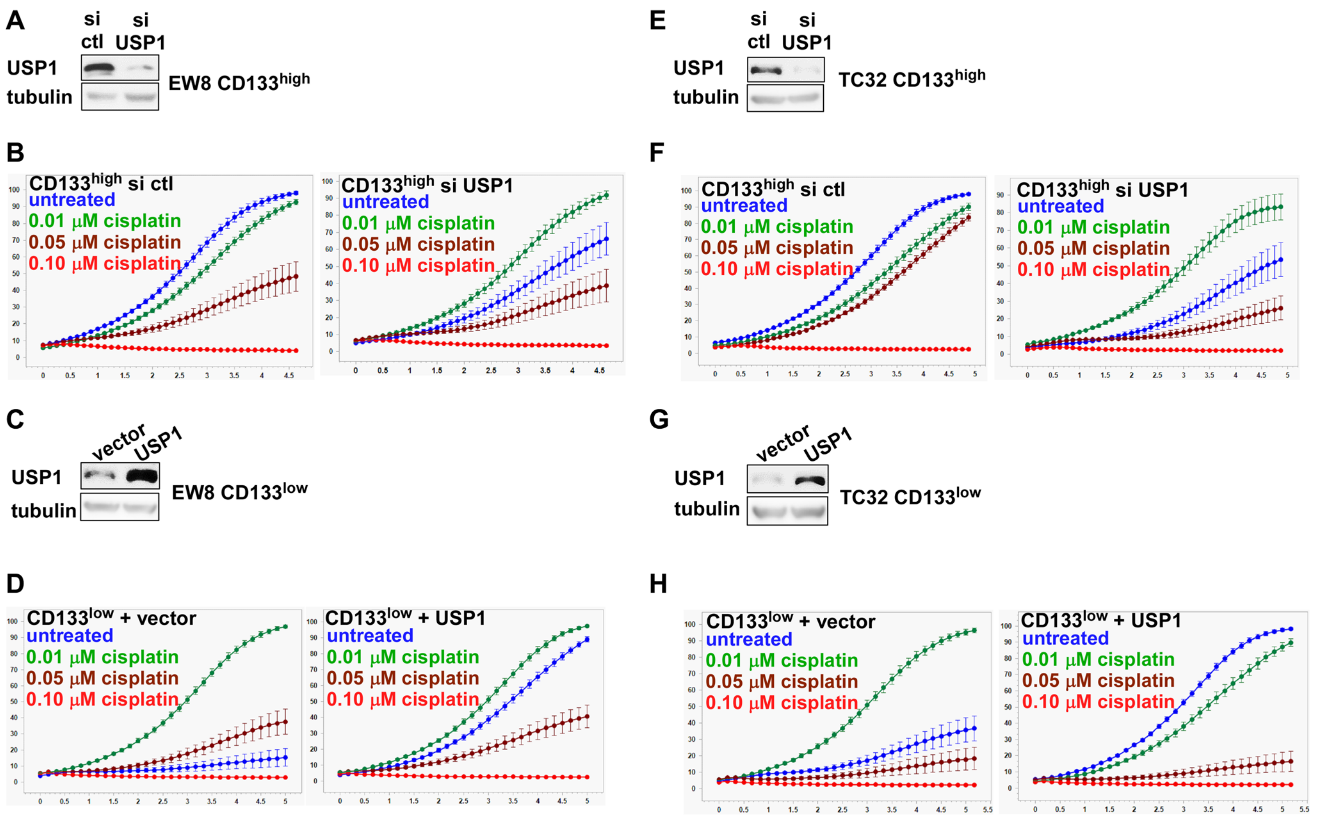

Supplementary Figure 1: USP1 determines chemotherapy sensitivity in Ewing sarcoma. (**A**) USP1 knockdown in EW8 CD133^{high} population. The CD133^{high} population derived from EW8 cells was transfected with control siRNA or USP1 siRNA and the USP1 protein levels were assessed by immunoblotting. (**B**) USP1 knockdown makes EW8 CD133^{high} population more resistant to chemotherapy. Cells in (A) were assessed for the sensitivity to indicated concentration of cisplatin using IncuCyte. (**C**) USP1 exogenous expression in EW8 CD133^{low} population. The CD133^{low} population derived from EW8 cells was infected with lentiviruses expressing USP1 or empty vector and the levels of USP1 were assessed by immunoblotting. (**D**) USP1 exogenous expression makes EW8 CD133^{low} population more sensitive to chemotherapy. Cells in (C) were assessed for the sensitivity to indicated concentration of cisplatin using IncuCyte. (**E**) USP1 knockdown in TC32 CD133^{high} population. The CD133^{high} population derived from TC32 cells was transfected with control siRNA or USP1 siRNA and the USP1 protein levels were assessed by immunoblotting. (**F**) USP1 knockdown makes TC32 CD133^{high} population more resistant to chemotherapy. Cells in (E) were assessed for the sensitivity to indicated concentration of cisplatin using IncuCyte. (**G**) USP1 exogenous expression in TC32 CD133^{low} population. The CD133^{low} population derived from TC32 cells was infected with lentiviruses expressing USP1 or empty vector and the levels of USP1 were assessed by immunoblotting. (**H**) USP1 exogenous expression makes TC32 CD133^{low} population more sensitive to chemotherapy. Cells in (G) were assessed for the sensitivity to indicated concentration of cisplatin using IncuCyte.

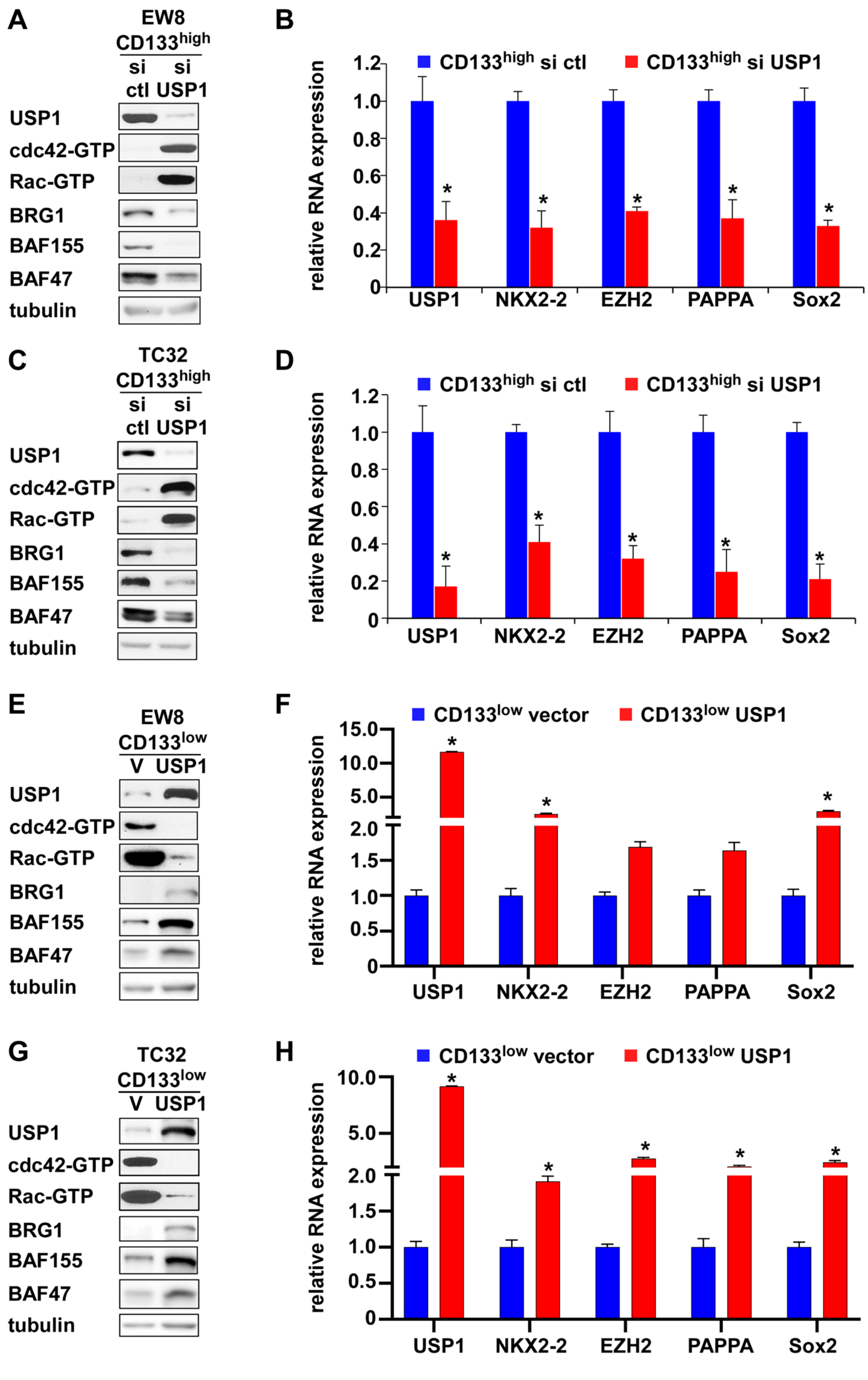

Supplementary Figure 2: USP1 inhibits cdc42 and enhances EWS-FL1 transcriptional output. (**A**) USP1 knockdown in EW8 CD133^{high} population results in activation of cdc42 and Rac and reduced BAF subunits. The levels of active cdc42 and active Rac were assessed by GST-PAK1 pulldown followed by immunoblotting. (**B**) USP1 knockdown in EW8 CD133^{high} population results in reduced EWS-FLI1 target gene expression. NKX2-2, EZH2, PAPPA, and Sox2 are EWS-FLI1 target genes. $^{*}p < 0.05$. (**C**) USP1 knockdown in TC32 CD133^{high} population results in activation of cdc42 and Rac and reduced BAF subunits. (**D**) USP1 knockdown in TC32 CD133^{high} population results in reduced EWS-FLI1 target gene expression. $^{*}p < 0.05$. (**E**) USP1 exogenous expression in EW8 CD133^{low} population results in inhibition of cdc42 and Rac and increased BAF subunits. (**F**) USP1 exogenous expression in EW8 CD133^{low} population results in increased EWS-FLI1 target gene expression. $^{*}p < 0.05$. (**G**) USP1 exogenous expression in TC32 CD133^{low} population results in inhibition of cdc42 and Rac and increased BAF subunits. (**H**) USP1 exogenous expression in TC32 CD133^{low} population results in increased EWS-FLI1 target gene expression. $^{*}p < 0.05$.

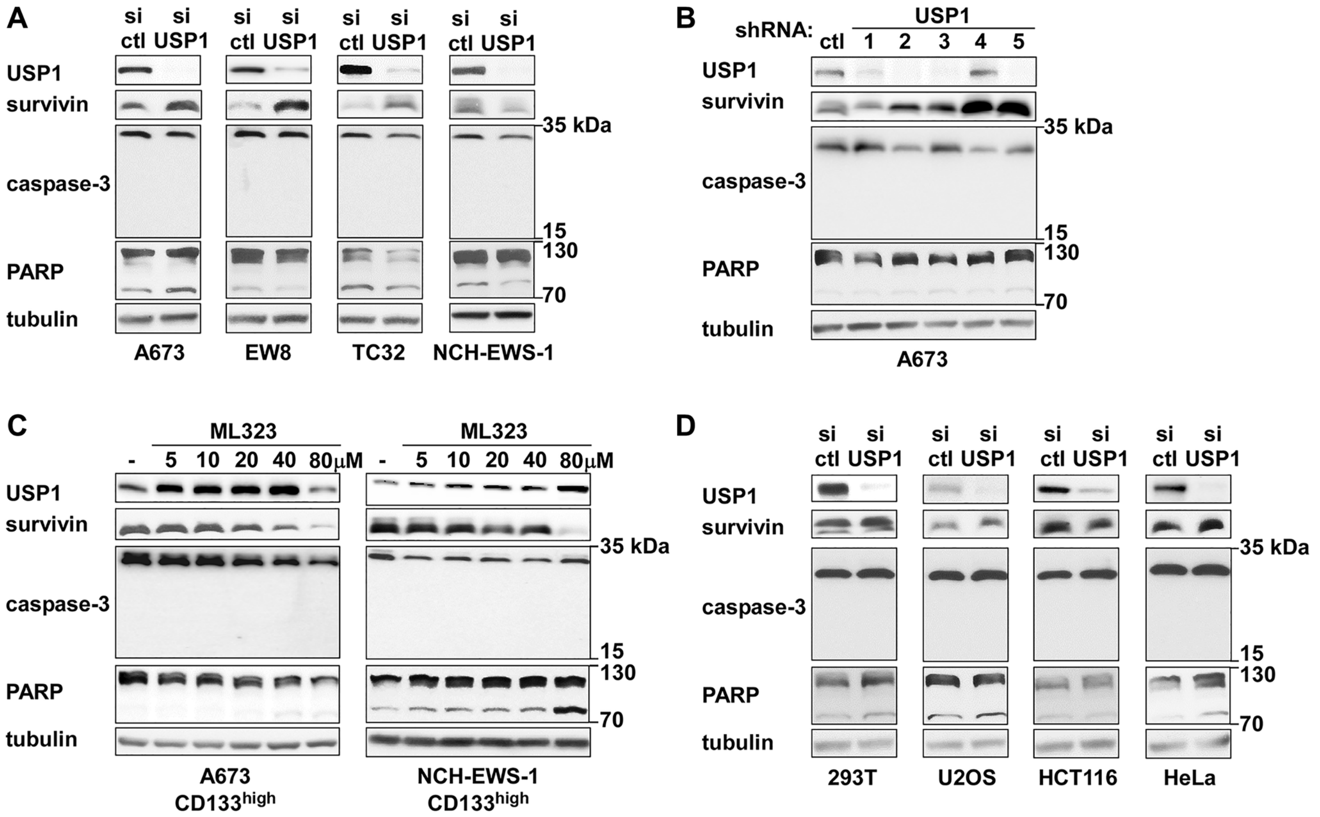

Supplementary Figure 3: The effects of USP1 manipulations on survivin protein levels and apoptosis markers. (**A**) The effect of USP1 siRNA knockdown on survivin and apoptosis markers in Ewing sarcoma cell lines. A673, EW8, TC32, and NCH-EWS-1 Ewing sarcoma cells were transfected with USP1 siRNA or control siRNA and the protein levels of USP1, survivin, caspase-3, and PARP were examined by immunoblotting. (**B**) The effect of USP1 shRNA knockdown on survivin and apoptosis markers in A673 cells. A673 cells were infected with lentiviruses expressing USP1 shRNA1-5 or control scrambled shRNA and the protein levels of USP1, survivin, caspase-3, and PARP were examined by immunoblotting. (**C**) The effect of ML323 on survivin and apoptosis markers in Ewing sarcoma cells. The $CD133^{high}$ population derived from A673 cells and NCH-EWS-1 PDX tumor cells were treated with the indicated concentration of ML323 for 24 hours and the protein levels of USP1, survivin, caspase-3, and PARP were examined by immunoblotting. (**D**) The effect of USP1 siRNA knockdown on survivin and apoptosis markers in non-Ewing sarcoma cells. 293T, U2OS, HCT116, and HeLa cells were transfected with USP1 siRNA or control siRNA and the protein levels of USP1, survivin, caspase-3, and PARP were examined by immunoblotting. The USP1 Western data in 293T, U20S, HCT116, and HeLa cells are the same as those in Figure 6A.
